# Errata

**Published:** 2012-06

**1. ****Iranian Journal of Microbiology** (2012) Mar; 4(1): 44–66.

**In the article titled: Development of a new DNA extraction protocol for PFGE typing of Mycobacterium tuberculosis complex**.

The correct names of authors are as follows:

Ghodousi A, Vatani S, Darban-Sarokhalil D, Omrani M, Fooladi A, Khosaravi A, Feizabadi MM

**2. ****Iranian Journal of Microbiology** (2012) Mar; 4(1): 40–43.

**In the article titled: Oral chromoblastomycosis: a case report**.

The correct names of authors are: Fatemi MJ, Bateni H

**3. ****Iranian Journal of Microbiology** (2011) Sep; 3(3): 112–7.

**In the article titled: Antimicrobial resistance profile and presence of class I integrongs among Salmonella enterica serovars isolated from human clinical specimens in Tehran, Iran**.

The correct names of authors are: Firoozeh F, Shahcheraghi F, Zahraei Salehi T, Karimi V, Aslani MM.

**4. ****Iranian Journal of Microbiology** (2010) Sep; 2(3): 165–7.

**In the article titled: Catalase-negative Staphylococcus aureus isolated from a diabetic foot ulcer**.

The correct names of authors are: Dezfulian A, Salehian M, Amini V, Dabiri H, Azimirad M, Aslani MM, Zali M, Fazel I.

